# 36-Year-Old Female with Catastrophic Antiphospholipid Syndrome Treated with Eculizumab: A Case Report and Review of Literature

**DOI:** 10.1155/2014/704371

**Published:** 2014-10-15

**Authors:** Marianna Strakhan, Mariana Hurtado-Sbordoni, Nahun Galeas, Kamila Bakirhan, Karenza Alexis, Tarek Elrafei

**Affiliations:** Department of Hematology and Oncology, Jacobi Medical Center Affiliate of Albert Einstein College of Medicine, 1400 Pelham Parkway, Bronx, NY 10461, USA

## Abstract

Catastrophic antiphospholipid syndrome (CAPS) is a rare but potentially life-threatening condition characterized by diffuse vascular thrombosis, leading to multiple organ failure developing over a short period of time in the presence of positive antiphospholipid antibodies (aPL). CAPS is a severe form of antiphospholipid syndrome, developing in about 1% of cases of classic antiphospholipid syndrome, manifesting as microangiopathy, affecting small vessels of multiple organs. It is acute in onset, with majority of cases developing thrombocytopenia and less frequently hemolytic anemia and disseminated intravascular coagulation. Lupus anticoagulant and anticardiolipin antibodies have been reported as predominant antibodies associated with CAPS. Treatment options often utilized in CAPS include anticoagulation, steroids, plasma exchange, cyclophosphamide therapy, and intravenous immunoglobulin therapy. Even though the reported incidence of this condition is considered to be low, the mortality rate is approaching 50%. The high rate of mortality should warrant greater awareness among clinicians for timely diagnosis and treatment of this life-threatening condition. Studies have shown that complement activation plays a key role in the pathogenesis of aPL mediated thrombosis in CAPS. We report a case of a 36-year-old female admitted with clinical and laboratory findings consistent with CAPS successfully treated with eculizumab, a terminal complement inhibitor.

## 1. Case Presentation

We would like to present a case of a 36-year-old female who was admitted to our hospital after being found in the bathtub with decreased responsiveness and profound weakness. The patient had no medical problems and had been in her usual state of health until one month before admission when she developed frequent headaches and blurry vision. Per family the patient had become more withdrawn recently and was not allowing anyone to visit her home. On examination in the Emergency Department, the patient was found to be hypertensive, to have blood pressure of 207/148 mmHg, to have heart rate of 110 beats per minute, and appeared confused and dehydrated, with multiple bruises over her body. Laboratory examination revealed white blood cell (WBC) count of 20.5/nL, hemoglobin of 12.3 g/dL, and platelets of 44,000/*μ*L. Patient's renal function was altered, presenting anion gap metabolic acidosis (suspected starvation ketosis) and respiratory alkalosis. Creatinine on admission was noted to be 7.0 mg/dL. Prothrombin time (PT) was 11.6 sec (reference range: 9.5–12.2 sec), activated partial thromboplastin time (aPTT) was 21.5 sec (reference range: 20.1–31.2 sec), and fibrinogen was 345 mg/dL. Mixing study was not performed at the time, since coagulation panel did not show prolongation of PTT. Troponins were elevated with no ST segment changes on electrocardiography compatible with non-ST segment myocardial infarction (NSTEMI). Ophthalmology was consulted and stated that visual loss and color blindness was likely secondary to intraretinal hemorrhage. A computed tomography (CT) of the brain was performed, which demonstrated numerous hypodensities in the frontal and parietal white matter. These changes were deemed to be acute as per radiological evaluation.

**Figure 1:**
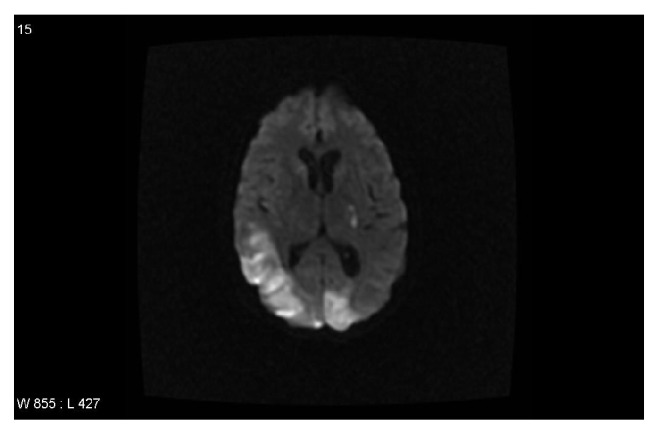
MRI brain on presentation.

**Figure 2:**
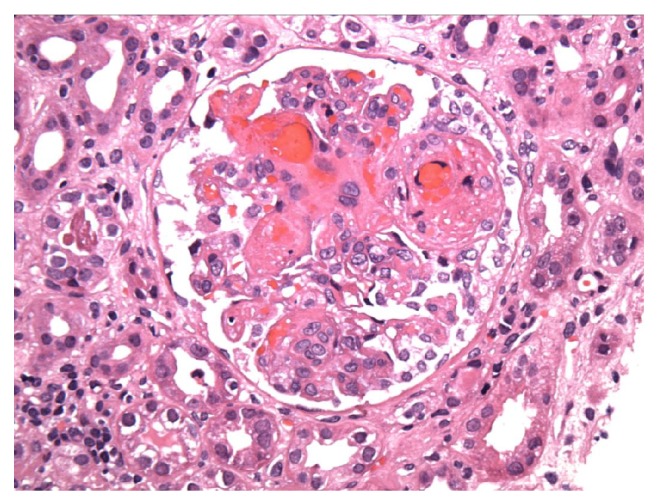
Kidney biopsy showing thrombotic microangiopathy.

Patient was admitted to the medical intensive care unit for close monitoring and blood pressure control. Further workup revealed lactate dehydrogenase levels (LDH) to be significantly elevated at 3,720 U/L and reticulocytosis of 4.2%. Repeat hemoglobin day after admission was noted to be 9.6 g/dL, which was deemed to be secondary to intravenous hydration of a previously dehydrated patient. Peripheral blood smear was significant for 5+ schistocytes per high power field with decreased absolute platelet count and large platelets, strongly suggestive of microangiopathic hemolytic anemia. These findings were concerning for thrombotic thrombocytopenic purpura, prompting initiation of plasmapheresis. After initial lack of response to plasmapheresis with fresh frozen plasma, patient was switched to cryosupernatant and received a total of 15 treatments of pheresis leading to improvement of her platelet count. Further hematologic workup revealed an ADAMTS13 of 58% (reference range: 68%–163%), eliminating thrombotic thrombocytopenic purpura as an etiology, and plasmapheresis was discontinued. Lupus anticoagulant (LA) drawn before initiation of plasmapheresis was reported as positive (18.6 sec, positive test: ≥8 second delta). Patient tested negative for cardiolipin antibody and beta-glycoprotein (<9 SGU). Workup for autoimmune disorder was unrevealing, demonstrating negative comprehensive antinuclear antibody (ANA) panel as well as normal complement levels. Malignant hypertension (HTN) was ruled out with negative metanephrines and renal ultrasound with no evidence of renal artery stenosis. Repeat LA testing was performed and was reported negative (6.6 sec) one month after first positive result. It was believed to have been a false negative result at the time as antiphospholipid antibodies may be removed with plasmapheresis. LA was repeated again at 7 weeks after plasmapheresis, at this point resulting positive (10 seconds), which was confirmed again several days afterwards. Magnetic resonance imaging of the brain (MRI) demonstrated infarction of the right parietooccipital region, left occipital polar region, and the right cerebellar hemisphere consistent with catastrophic antiphospholipid syndrome (Figure 1). Repeat MRI was notable for petechial hemorrhagic conversion of infarct and anticoagulation was deferred at this time as per neurology recommendations. Patient was started on hemodialysis due to her persistent renal failure. Renal biopsy was performed reporting evidence of both acute and chronic thrombotic microangiopathy involving both glomeruli and arteries/arterioles favoring diagnosis of antiphospholipid antibody syndrome (Figure 2). Patient's clinical features were consistent with CAPS (including multiple strokes, non-ST elevation myocardial infarction, end stage renal disease, intraretinal hemorrhage, renal biopsy with multiple arterial thrombi, and positive lupus anticoagulant).

At this time standard therapy for catastrophic antiphospholipid syndrome was initiated, such as pulsed dose steroids. Patient was subsequently maintained on prednisone. Unfortunately, patient was not clinically improving despite plasmapheresis and prednisone. Her creatinine remained elevated and visual acuity was not improving. Given concern regarding use of cyclophosphamide in setting of acute renal failure, it was withheld at that time. We have performed review of the literature, which was notable for several case reports of successful outcomes with addition of eculizumab to standard therapy in CAPS.

After a thorough discussion with patient and family in regard to the risks and benefits of eculizumab, therapy was initiated. Patient was immunized against* Neisseria meningitidis* and* Streptococcus pneumoniae* prior to initiation of eculizumab. At this time eculizumab was administered 900 mg intravenously weekly × 4 weeks, followed by 1200 mg at week 5 and then continued every 2 weeks thereafter. Patient was then transferred to a rehabilitation ward, where she underwent acute intensive therapy and continued to receive hemodialysis three times weekly. She was slowly tapered off prednisone and currently remains off steroid therapy. Anticoagulation was readdressed once imaging revealed resolution of hemorrhagic focus in the brain. Patient was initiated on subcutaneous heparin while evaluating for bleeding. However, with a subtherapeutic dose of heparin, patient was noted to develop an elevated partial thromboplastin time (PTT) level >100 seconds. Patient previously had normal PTT levels; however, despite multiple attempts at reinitiating unfractionated heparin (UFH), her PTT remained supratherapeutic, without bleeding manifestations. PTT normalized after cessation of subcutaneous heparin. Repeat LA testing was performed at the time (7 weeks from initial testing) which resulted in positive test of 10 seconds. DRVVT was performed at the time which resulted with ratio of 1.15 (normal is less than or equal to 1.15). It is important to note that patient was receiving prednisone at the time.

**Figure 3:**
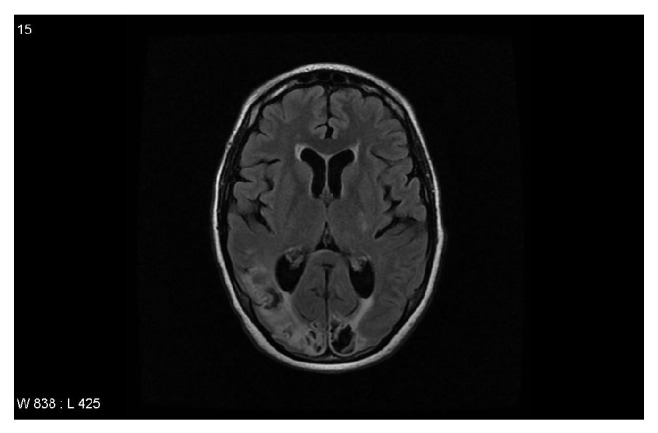
MRI brain 5 months after presentation.

Anticoagulation was subsequently discontinued and patient remained on hemodialysis and eculizumab. Patient and family refused further attempts at anticoagulation until 5 months after initial presentation when she successfully underwent anticoagulation with UFH being able to maintain therapeutic PTT. She was subsequently initiated on coumadin therapy, which she remains on to date. MRI of the brain was repeated 5 months after presentation revealing no acute changes with evidence of chronic basal ganglia infarcts and extensive encephalomalacia (Figure 3).

Patient currently has improved significantly since her initial presentation. Her vision has improved allowing her to see shades of light and shapes and she has not had any further thrombotic episodes on clinical presentation and imaging. She is now able to ambulate, which she was unable to do at presentation. Her LDH has normalized to 177 U/L, hemoglobin remains at 13.5 g/dL, and platelets are 514,000/*μ*L. Lastly, her creatinine has now improved greatly from 7.0 mg/dL on presentation to 1.6 mg/dL, and patient has been successfully taken off hemodialysis. Of particular note is that improvement in patient's symptoms, visual acuity, and functional status as well as LDH and creatinine have occurred after eculizumab was initiated prior to receiving anticoagulation.

## 2. Discussion

APS is a systemic autoimmune disorder characterized by arterial and/or venous thrombosis and recurrent fetal loss and can be associated with thrombocytopenia [1]. It was first recognized in patients with systemic lupus erythematous (SLE) and later found in association with other autoimmune disorders. This condition has also been recognized as a syndrome that can develop independent of any underlying disease, known as primary APS [1]. CAPS, a fatal variant of APS, with a prevalence of 1% of APS population, was first described in 1992 and defined as thrombosis of at least three different organ systems over a very short period of time with histopathologic evidence of multiple small vessel occlusions and high titers of antiphospholipid antibodies (aPL) [1–5].

Given the rarity of CAPS, our ability to analyze and study it in a systematic way has been challenging. Therefore, a registry was created in 2000 by the European Forum, which compiled all published case reports and newly diagnosed cases from all over the world. This registry can be freely consulted at http://www.med.ub.es/MIMMUN/FORUM/CAPS.HTM. Currently, the CAPS registry documents the entire clinical data of 280 patients whose clinical, laboratory, and management data have been recorded. An analysis was done thereafter from this registry of patients with CAPS describing the clinical and laboratory features, precipitating factors, therapies, and outcomes [6]. In this CAP study analysis, gender was commonly found to be females (72%) with a mean age of 37 ± 14 years and with a majority suffering from primary APS (40%) as opposed to SLE, lupus-like disease, and other autoimmune diseases. Interestingly it was also found that CAPS was the first manifestation of APS in 46% of the 280 patients. Triggering factors that lead to the development of CAPS were commonly noted to be infections, surgery, withdrawal of anticoagulation, medication, obstetric complications, neoplasia, and lupus flare [6, 7]. (See Table 1.)

The clinical manifestation of CAPS depends on the organ involvement affected by thrombosis. The major organs involved during the catastrophic episode were renal (71%), followed closely by lung (64%), brain (62%), heart (51%), and skin (50%) [6]. (See Table 1.) Our patient presented with renal failure, cerebrovascular accident, NSTEMI, and severe hypertension consistent with prior analysis of clinical manifestations and organ involvement in patients with CAPS. Interestingly, our patient's hypertension was initially worked up as a possible etiologic source for our patient's severe disease manifestation. However, it has been reported and also implemented in the preliminary criteria for classification of CAPS that hypertension tends to occur in conjunction with renal involvement [5]. Since 1998, in the study of 50 patients with CAP, it was noted that thirty-nine (78%) of patients had renal involvement commonly accompanied by hypertension that was often malignant [4]. Furthermore, renal thrombotic events were demonstrated in most of the patients with kidney biopsy showing small vessel occlusive disease and thrombotic microangiopathy [4]. Our patient's kidney biopsy also showed thrombotic microangiopathy a pathologic hallmark of CAPS.

Laboratory findings in CAPS patients may include thrombocytopenia, hemolytic anemia which is often accompanied by schistocytes, and disseminated intravascular coagulations (DIC) [6]. The autoantibodies of interest to diagnose APS are anti-B2-glycoprotein I detected by enzyme-linked immunosorbant assay (ELISA), anticardiolipin (aCL), or lupus anticoagulant (LA) assay [1, 8]. The recent 2006 revised classification criteria for APS updated the timeframe for the presence of elevated titers of antiphospholipid antibodies from >6 weeks to its persistence for >12 weeks [8, 9]. Our patient demonstrated the presence of LA antibodies upon admission and seven weeks after being hospitalized despite undergoing dialysis and plasmapheresis with transient loss of antibody. Review of the literature was unrevealing regarding length of time it may take to develop recurrent positive antibody testing after plasmapheresis. Given that our patient has lost the antibody (LA delta 6.2–6.6 seconds) transiently after plasmapheresis, we would like to add to the world of literature that it may take 7 weeks to note a recurrence in the elevated titer of LA. It may also be worth researching this timeline further as repeating antibody testing prior to 12 weeks may be sufficient.

Laboratory studies have become important diagnostic criteria for detecting APS. LA activity is detected by coagulation assays that adhere to guidelines from the International Society of Thrombosis and Haemostasis (ISTH), updated in 2009 by Pengo et al. which include (a) prolonged phospholipid-dependent coagulation time found on a screening test (activated partial thromboplastin time and dilute Russell's viper venom time), (b) failure to correct prolonged coagulation time during mixing studies, (c) correction of prolonged coagulation time found on screening test by adding excess phospholipids, and finally (d) exclusion of other coagulopathies [1, 10]. The screening test criteria cutoff in the updated ISTH guidelines includes cutoff value above 99th percentile of the distribution [10].

Lupus anticoagulant was tested in our institution using STACLOT LA 20 reagent system, designed for qualitative detection of LA in plasma by use of hexagonal phase phosphatidylethanolamine (HPE). STACLOT assay uses a phospholipid-poor thromboplastin designed to be LA sensitive. The LA test procedure is based on the following principle: the test plasma that is suspected to contain LA is first allowed to incubate at 37°C with HPE and without it. Next, APTT is performed on both tubes using a LA sensitive reagent; if LA is present in the test plasma, it would be neutralized by HPE, and this would result in a shortening of the clotting time compared with that of the tube without HPE. By comparing the difference between the two clotting times, the presence of LA antibodies in the test plasma can be identified. This test is capable of distinguishing these LA antibodies from antifactor antibodies, factor deficiencies, and heparin, since all four conditions may cause a prolongation of the APTT test.

A cutoff of delta ≥8 seconds has been determined as a positive result. Cutoff for assay is 20G/M PL as is the standard. It is reported that both activated partial thromboplastin time based assays and dilute Russsell's viper venom time assay are appropriate for LA and one positive test suffices LA positivity [9].

Differential diagnosis for CAPs is challenging as sepsis, thrombotic thrombocytopenic purpura (TTP), hemolytic uremic syndrome (HUS), and disseminated intravascular coagulation (DIC) all share similar features [3]. However, the differences between these entities can be worked up to properly exclude them as a diagnosis. Patients with sepsis and CAPS share the symptoms of systemic inflammatory response syndrome (SIRS) and being that infection is a most common triggering factor for CAPS they can coexist. TTP shares similar features of thrombocytopenia, hemolytic anemia, schistocytes, renal dysfunction, and neurological features, but they do not tend to have positive antiphospholipid antibodies [3]. Our patient's differential diagnosis on admission included TTP; however, it was noted that her ADAMTS13 activity was >5% and thus not consistent with TTP and LA was positive. The diagnosis of CAPS from other microangiopathic syndromes can be very challenging because of the acute onset of thrombosis leading to multiple organ failure; therefore, early diagnosis and management are imperative for patient survival.

Treatment guidelines for CAPS include a combination of anticoagulants (AC), corticosteroids (CS), intravenous immunoglobulins (IVIG), plasma exchange (PE), and cyclophosphamide [6, 11]. However, new therapeutic modalities have emerged for the treatment of CAPS, especially in cases of refractory CAPS. These treatment modalities include rituximab, defibrotide, and eculizumab [12]. Rituximab, a chimeric monoclonal antibody against CD20 surface antigen on B cells is approved for the treatment of CD20+ B cell non-Hodgkin lymphoma and for rheumatoid arthritis. Rituximab has also been reported in the management of patients with severe, refractory SLE [12, 13]. Berman et al. [13] reviewed the CAPS registry and identified 20 patients treated with rituximab in which 75% of these patients recovered from CAPS and 20% died during the catastrophic event. Given the results from that review it was noted that rituximab could play a role in treatment of aPL positive patients and those with refractory CAPS, but its effectiveness could not be isolated to rituximab alone since patients received combined therapy with AC, CS, PE, and/or IVIG. Furthermore, the patient sample size was too small to draw well-founded conclusions [13]. Defibrotide, another treatment option, is a mixture of single stranded and double stranded phosphodiester oligonucleotide derived from porcine mucosa DNA and its mechanism of action is to diminish formation of clots by upregulation of prostacyclin and prostaglandin E2, reduction of leukotriene B4, inhibition of monocyte superoxide anion generation, expression of thrombomodulin on vascular endothelial cells, and alteration of platelet activity [12]. According to Espinosa et al. [12] defibrotide was reported in the use of two patients with CAPS. The first patient who initially presented with thrombocytopenia, proteinuria, and soft tissue necrosis of his digits had a good outcome, but the second who initially presented with renal failure complicated by intestinal and cutaneous thrombosis had an unfortunate outcome leading to death. In the CAPS registry study of 280 patients it was noted that recovery was more frequent in those treated with anticoagulants, but given that most patients received combination therapy, the highest recovery rate was achieved by combination of AC, CS, in addition to PE and/or IVIG. Cyclophosphamide in this study did not demonstrate much benefit, but this is probably due to its use in the most severe cases of CAPS [6]. We have decided to forgo the use of cyclophosphamide given the severity of our patient's acute renal failure and concern for renal toxicity of the therapy.

Although we have attempted to utilize anticoagulation in our patient, we have noted a significantly prolonged PTT with use of subcutaneous unfractionated heparin. Etiology of such response is unclear and review of the literature did not reveal similar cases. Repeat LA testing at the time revealed return of positive antibody with delta of 10 seconds. Patient remained on prednisone at the time possibly contributing to a decrease in the antibody titer despite its recurrence.

Given our patient's lack of response to standard therapy and inability to anticoagulate, our review of the literature revealed four publications in the successful management of CAPS with use of eculizumab [14–17]. Eculizumab is currently approved by the Food and Drug Administration (FDA) for the treatment of paroxysmal nocturnal hemoglobinuria [12] and has also recently been FDA approved for the treatment of atypical hemolytic uremic syndrome [18]. We would like to update the literature database of another case report of CAPS treated with eculizumab.

Several mechanisms have been proposed for the pathogenesis of catastrophic APS such as molecular mimicry, infections, activation of endothelium microvasculature, and small vessel occlusions resulting in SIRS and release of inflammatory cytokines, products of complement (C3, C5) who in combination with aPL antibodies have led to thrombosis characteristic of CAPS [3]. Although the underlying mechanisms of the pathogenesis of CAPS continues to unravel, many theories have proposed that uncontrolled activation of the complement system is a major factor by which aPL antibodies induce tissue injury [8]. In mouse models, APS was induced by passive transfer of aPL-IgG antibodies and it was found that complement blockade at C3 by using Crry-Ig, an exogenous inhibitor of C3, prevented fetal loss, growth retardation, and tissue injury in these mouse models [19]. Furthermore, in another study of mouse models, it was shown that C5a-C5aR interaction was a critical effector of aPL antibody induced tissue injury and that C5aR deficiency, C5aR antagonist, and anti-C5 monoclonal antibodies inhibited mediators and effectors of tissue injury and prevented the deleterious effects of aPL antibodies [20]. Another study also demonstrated that C6 deficient mice had markedly reduced platelet-leukocyte aggregates and thrombotic occlusion. Animals that were again treated with anti-C5 antibody prevented the prothrombotic activity of aPL antibodies supporting the fact that terminal complement is a key pathogenic implicator in the mechanism of aPL antibody-mediated thrombosis, suggesting ultimately that anti-C5 antibodies could be useful in treating patients with CAPS [21].

Since multiple studies have shown that complement activation plays a key role in the pathogenesis of aPL antibody-mediated thrombosis, the use of a terminal complement inhibitor eculizumab has been reported as a novel therapy in few case reports of antiphospholipid syndrome thus far in the literature [8]. The first case study reporting the use of eculizumab for treating CAPS reported by Lonze et al. [15] was of a patient whose primary CAPS had led to infarction of his liver, spleen, heart, and kidneys. Given that the patient's renal function never recovered, he was referred for renal transplantation and received eculizumab as prophylaxis to prevent thrombotic microangiopathy in CAPS [15]. Following this, a study reported by Shapira et al. [14] demonstrated sustained remission in recurrent thrombotic events with eculizumab therapy for >3 years of a young man with CAPS who had been resistant to other standard interventions. Given these findings and the beneficial outcome of using eculizumab, a case series was reported of three patients who received eculizumab after renal transplant with demonstrated improvement of thrombotic microangiopathy due to APS [16]. Interestingly, a follow-up case series by Lonze et al. [17] who reported the first case report of using eculizumab for CAPS prior to renal transplant has recently been noted in the literature. This case series consisted of three patients with APS, two who also had a history of CAPS that were treated with systemic anticoagulation along with eculizumab. Use of eculizumab in these patients prior to and after renal transplantation with an average followup ranging from 4 months to 4 years thus far showed successful outcome of functioning renal allografts. These published case reports again reinforce the fact that eculizumab is a promising agent for prevention of CAPS.

## 3. Conclusion

The importance of investigating complement inhibitors for treating patients with CAPS certainly deserves clinical attention. We would like to report another case of successful management of CAPS with use of eculizumab, thus far showing a promising recovery in a known fatal disease. Eculizumab was used in our patient during the initial course of her disease, without heparin. Furthermore, it is important to also acknowledge that heparin has inhibitory mechanism against complement activation based on mouse models [11]. Our patient's successful course during her initial presentation could be attributed to beneficial role of eculizumab and its activity against complement activation. This occurred without much additional benefit of heparin due to initial adverse reaction to the drug. With our patient's continued success to recovery, it is important to emphasize the role of complement inhibitors as direct-targeted therapy in CAPS. This case report again emphasizes the importance of complement inhibitors such as eculizumab for the future management, treatment, and prevention of CAPS.

## Figures and Tables

**Table 1:** Precipitating factors and clinical manifestations of 280 patients with catastrophic APS from the CAPS registry.

Precipitating factors	Number of patients	(%)
Infection	62	(22)
Surgery	28	(10)
Oral anticoagulation withdrawal/low INR	22	(8)
Medications (captopril, oral contraceptives, danazol, and thiazide diuretics) [7]	20	(7)
Obstetric complications	19	(7)
Neoplasia	14	(5)
SLE flare	8	(3)

First clinical manifestation of the catastrophic episode		
Pulmonary involvement	67	(24)
Neurological involvement	50	(18)
Renal involvement	49	(18)
Cutaneous involvement	28	(10)
Cardiac involvement	27	(10)
Adrenal involvement	3	(1)

Organ involvement during the episode		
Kidney	180	(71)
Lung	163	(64)
Brain	158	(62)
Heart	131	(51)
Skin	128	(50)
Liver	85	(33)
Gastrointestinal	60	(25)
Peripheral venous thrombosis	59	(23)
Spleen	48	(19)
Adrenal glands	33	(13)
Peripheral artery thrombosis	27	(11)
Pancreas	19	(8)
Retina	17	(7)
Peripheral nerve	12	(5)
Bone marrow	10	(4)

Reproduced with permission from Cervera et al. [6].
